# Informed consent for research obtained during the intensive care unit stay

**DOI:** 10.1186/cc5120

**Published:** 2006-12-08

**Authors:** Catherine Chenaud, Paolo Merlani, Samuel Luyasu, Bara Ricou

**Affiliations:** 1 Service of Intensive Care, Department of Anesthesiology, Pharmacology and Intensive Care, University Hospital of Geneva, Rue Micheli-du-Crest 24, 1211 Geneva 14, Switzerland

## Abstract

**Introduction:**

Patients in the intensive care unit (ICU) may be in an inadequate condition to give their informed consent for research. The aim of this study was to analyse the ability to recall participation in a clinical trial for which ICU patients had given their consent.

**Methods:**

The data presented are a two-step observational study: first, a protocolled informed consent procedure was conducted then the informed consent was given by the patient, and second, a patient interview was held 10 ± 2 days later by the same investigator. The primary endpoints were the ability to recall their participation in the clinical trial, as well as its purpose and related risks. As secondary endpoints, we investigated whether asking questions about the clinical trial or reading the informative leaflet was related to the recall. To be included in the study, the patient had to have a Glasgow Coma Scale score of 15, be fully oriented and free of mechanical ventilation, and be judged competent by both the investigator and the attending physician. Patients admitted to the ICU after major surgery or trauma were eligible. However, patients who refused to participate, or those whose next-of-kin gave consent, were excluded.

**Results:**

Of the 44 patients, 35 (80%) recognized, 10 to 12 days after informed consent had been obtained, that they had participated in the clinical trial, but only 14 out of 44 (32%) could recall the clinical trial purpose and its related risks. More patients with complete recall had read the informative leaflet or asked at least one question before signing the informed consent. Asking at least one question was associated with complete recall.

**Conclusion:**

Our results confirm that obtaining informed consent for research during an ICU stay is associated with poor patient recall of participation in a clinical trial and its components (purpose and risk). Whether encouraging reading the informative leaflet and asking questions about the clinical trial improves the informed consent procedure remains to be fully investigated.

## Introduction

Within the past few decades, medicine, especially critical care medicine, has progressed spectacularly as a result of medical research and the participation of patients in clinical trials. The Declaration of Helsinki and international guidelines require that the patient give informed consent before participating in a study. This informed consent is essential to respect the autonomy of patients and protect them against abuse [1-3].

Therefore, to respect the ethical principles of clinical research, informed consent for research demands to be held at a high standard because the patient does not always benefit directly from the research results and might suffer some risks for the good of the community. Keeping with this high standard, informed consent must fulfil three mandatory conditions as described in the Belmont report: adequate information about the study with a complete disclosure of the risks and benefits, the patient's comprehension, and the voluntariness [4-7]. This means that the patient should be able to freely decide their participation in the study without any external pressure and that they could resign from the study at any time without any consequence to their care. To respect this requirement and the principle of autonomy, it is essential that patients comprehend and recall knowledge of the study components as well as their participation in the study. However, patients in the intensive care unit (ICU) may be in an inadequate condition to give their informed consent for a research study because their capacity for comprehension is questionable. However, lighter sedation of ICU patients permits an increasing number of them to remain conscious. These patients seem to be able to understand the information presented and to decide freely if they would like to participate in the study. However, there is no available consensus on the capacity required to give consent [8]. As objective criteria regarding the decision-making capacity of patients are lacking in international and national directives, investigators usually use their clinical judgement and the Glasgow Coma Scale (GCS) as guidance. Previously, we showed that 22% of clinical trial participants could not recall their participation in the clinical trial despite the fact that informed consent was obtained in an ideal situation, namely before their admission to the ICU [9]. Furthermore, 25% of patients were unable to recall the clinical trial purpose and its related risks. In view of these results, we hypothesized that informed consent for research obtained from a patient during their ICU stay is associated with an even worse ability for them to recall their participation in a clinical trial and its components (purpose and risk).

## Materials and methods

### Design

This investigation on informed consent was an observational substudy of a clinical trial about inflammation, described elsewhere [10]. The present study was designed in two steps: first, a protocolled informed consent procedure was conducted then the informed consent was given by the patient, and second, a patient interview was held 10 ± 2 days later by the same investigator about their participation, the purpose, and the risks related to the clinical trial on inflammation.

### Setting

This study was performed in a 20-bed surgical ICU of a tertiary-university-affiliated teaching hospital receiving 1,600 patients per year.

### Type of participants

Patients admitted to the ICU after a major surgery or trauma were eligible. To give their consent to participate in the clinical trial on inflammation, patients had to present with a GCS score of 15, be fully oriented and free of mechanical ventilation, and judged competent by both the investigator and the attending physician. Patients who refused to participate, met any of the exclusion criteria for the clinical trial on inflammation, and those whose next-of-kin gave consent were not considered for the present study. Furthermore, incompetent or non-French-speaking patients, and those with psychiatric disorders, senile dementia or other intellectual disabilities were not included.

### The informed consent procedure

A protocoled procedure detailing how to obtain informed consent was developed to ensure that the two investigators contributing to the study obtained consent in an identical manner. The investigators were physicians who were not caring for the patients enrolled in the study. Informed consent was obtained on the ICU admittance date, after a 20-minute individual oral presentation. During this presentation, the investigator explained the clinical trial on inflammation, emphasizing two clinical trial components: the purpose and its related risks. The defined keyword for the clinical trial purpose was inflammation. The clinical trial risk for the patient was that 10 ml of their blood would be drawn daily for the first five days of their ICU stay, and a final blood draw would be required at day 28. The investigator told the patient that the risk was minimal.

The patient then received a one-page informative leaflet. At the end of this procedure, the investigator asked the patient if he had any questions about the clinical trial before signing the informed consent form. The investigator noted whether the patient asked any questions and/or if he read the informative leaflet in his presence. These two attitudes of the patient (asking one or more questions and reading the informative leaflet) were defined *a priori *in the consent procedure. No other attitudes of the patient were recorded or tested.

This informed consent procedure conforms to recommendations on the ethical conduct of clinical research involving patients in the ICU [11].

### Data collection

Patient age, gender, history of daily alcohol intake type of admission and diagnosis, as well as Simplified Acute Physiological Score second version (SAPS II) [12] were recorded on admission to the ICU. The lengths of mechanical ventilation and ICU stay were also noted.

When informed consent was obtained, clinical and laboratory values, the Sequential Organ Failure Assessment (SOFA) score [13], and medical treatment given within the 24 hours before and after the consent procedure were assessed.

At 10 ± 2 days (range), the investigator met the patient to plan the blood sampling to be performed on day 28. At this time, the investigator assessed whether the patient could recall participation in the clinical trial and/or any of the clinical trial components.

The primary endpoints were the ability of the patient to recall participation in the clinical trial, as well as the purpose and related risks of the clinical trial. As secondary endpoints, we investigated whether asking questions about the clinical trial or reading the informative leaflet was related to the recall.

Patients who could report their participation in the clinical trial on inflammation, the clinical trial purpose and the related risks were assigned to the 'complete recall' group. Patients lacking one or more components were assigned to the 'incomplete recall' group.

### Ethical issue

The clinical trial on inflammation itself, as well as the informative leaflet and the consent form, were approved by the Ethical Committee for Human Research of our institution. Specific informed consent had not been sought for this study. The educational status of the patient, that was the sole data not available in the dataset of the clinical trial, was subsequently retrieved from the administrative file.

### Statistical analysis

StatView for Windows version 5.0.1 (SAS Institute Inc., Cary, NC, USA) was used for statistical analysis. Patients were stratified as having either complete or incomplete recall; these data were compared separately by using a two-tailed Fisher's exact test, an unpaired *t *test or a Mann–Whitney *U *test, as deemed appropriate. We also assessed the sensitivity, specificity, the positive predictive value, the negative predictive value and the likelihood ratio of factors that were statistically significant on the basis of the univariate analysis performed to predict complete recall of the clinical trial. Odds ratios with 95% confidence intervals were calculated to estimate the effect size of risk factors associated with complete recall. All tests were two-tailed; *p *< 0.05 was considered significant.

## Results

**Figure 1:**
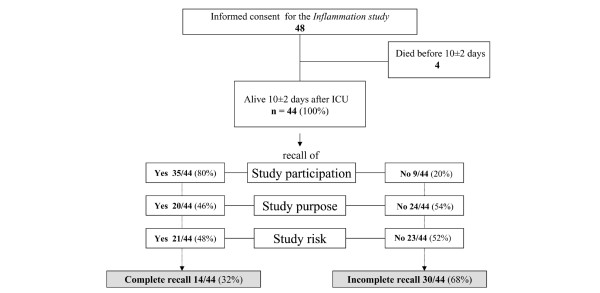
Distribution of the patients.

Between May 2000 and January 2001 we included 48 patients, of whom four died during the ICU stay (Figure 1). We therefore analysed the data on 44 patients who signed the informed consent and were alive at 10 ± 2 days; at this time all patients presented with a GCS score of 15, were fully oriented and were judged competent by the investigator.

Of the 44 patients, 35 (80%) recognized they had participated in a clinical trial; 14 of the 44 patients (32%) could recall their clinical trial participation as well as the clinical trial components, namely the clinical trial purpose and the related risk (Figure 1). These 14 patients were assigned to the 'complete recall' group.

Patients with complete recall did not differ from patients with incomplete recall with regard to their demographic, educational, and admission characteristics (Table 1). The lengths of mechanical ventilation and ICU stay were similar in both groups.

When informed consent was obtained, the clinical and laboratory values of the patients did not differ between the two groups (Table 2). The medications administered during the 24 hours before and after the informed consent were similar in the two groups (Table 2). More patients with complete recall had read the informative leaflet or had asked at least one question before signing the informed consent (13 out of 14 (93%) versus 18 out of 30 (60%); *p *= 0.03). Asking at least one question was associated with a complete recall of the clinical trial (*p *= 0.03). The sensitivity, specificity, positive predictive values, and negative predictive values of 'asking one question' and 'reading the informative leaflet or asking one question' to differentiate patients with complete recall from patients with incomplete recall are shown in Table 3.

The first investigator included 15 (34%) patients in the clinical trial during the first three months of the inclusion period, and the second investigator added 29 (66%) patients during the last six months. Patient characteristics, attitude during the informed consent procedure, and rates of recall did not differ significantly between investigators (Table 4).

## Discussion

A great majority of ICU patients who had consented to participate in a clinical trial during their ICU stay recalled their clinical trial participation after 10 ± 2 days. Other studies, including those on patients with acute myocardial infarction, have reported clinical trial participation recall rates similar to ours [14-16]. Our rate is also similar to the rate we found in a study in which informed consent was obtained in an ideal situation, namely before ICU admission [9].

Although this may seem very encouraging, only 32% of the patients who gave consent during their ICU stay recalled clinical trial components; this is in contrast to our 'ideal situation' previous study in which 75% of patients had a complete recall [9]. The low rate of complete recall in the present study could be explained by the difficulty of some ICU patients to process information given the stress of the acute phase and possibly experiencing feelings of dependence and anguish [17]. Our routine clinical evaluation might not detect this potential cognitive defect [18]. The reasons why some patients are able to recall whereas others cannot therefore remain unclear.

The severity of disease, the neurological status of the patients, and the medications received in the ICU when informed consent was obtained and during the 24 hours after the informed consent procedure were similar in both groups of our patients.

Inadequate information disclosure to some patients that could explain our results can reasonably be excluded because the information procedure was well standardized. In contrast with previous reports, we found that neither age nor educational status influenced the ability to recall clinical trial components [19,20]. This might reflect the fact that the information presented was not difficult to understand.

If the informed consent satisfies the three criteria specified by international guidelines, the consent is deemed valid [1,2,21]. However, if the clinical trial has already begun and the patient is unaware of the purpose, related risks, and their participation in the clinical trial, they are obviously unable to decide mindfully whether to continue in the clinical trial or to withdraw from it at any time. In this case, we might question the respect of the participant's autonomy offered by the informed consent. This leads us to view the informed consent as a process rather than a simple procedure. The informed consent process requires, to our mind, multiple conversations on several occasions while the research is conducted.

We found simple but important factors associated with complete recall of the clinical trial components. We tried to identify factors that were easy to observe by an investigator such as 'asking questions' and 'reading the informative leaflet'. In our previous study, we found that more patients who were able to mention all clinical trial components had read the leaflet and had asked at least one question. 'Asking questions' increased the chance of recall in critically ill patients. We could speculate inversely that asking no questions could increase the potential for poor initial comprehension of the clinical trial components.

Our results are in line with Flory and Emanuel's findings [22], which concluded that person-to-person interaction with clinical trial participants may be the most effective way of improving their understanding.

This finding revives the debate about informed consent for research in the ICU. In the past, deferred consent [23] or waiving of consent for research in the emergency setting [24,25] was considered acceptable in particular research situations. However, during the past few years, legislation has tried to enhance protection for incompetent patients. Barriers to the inclusion of ICU patients in research studies have increased, especially across Europe [2,26]. We fully support the idea that oversight is necessary to ensure the uniform application of ethical standards [11], but the most dependable safeguard for the research subject is investigator understanding and respect of the ethical requirements of clinical research.

Our study presents some limitations. First, the small number of patients enrolled was dependent on the clinical trial. This might have impeded the detection of other potential factors that could influence the recall of the clinical trial. In addition, the small clinical trial size precluded the possibility of a multivariate analysis. Second, we did not investigate the recall of patients who had refused to participate in the clinical trial on inflammation primarily because there was no consent to investigate. Perhaps the degree of recall might have been different in patients who had refused participation. Third, we did not assess patients' cognitive capacity. There is good evidence that the cognitive capacity of ICU patients, or even sick patients in general, is impaired [27,28]. However, spending more time with patients to test their cognitive capacity, and interacting with them for much longer than for a 'standard' informed consent procedure, would have biased our results. It is for this same reason that we did not measure the patients' memory. Fourth, scores of severity of illness and laboratory values may suggest that patients with incomplete recall were more ill than those with complete recall. Although not statistically significant, these results do not allow for the possibility that the severity of the disease might act on recall. However, the fundamental message remains that a poor rate of recall of a clinical trial is present in ICU patients. Finally, as our patients were presumed fully competent, the study faced the best possible situation in ICU patients. We are aware that these patients do not represent the majority of ICU patients and our results may therefore not be generalizable. However, it is reasonable to speculate that the rate of recall may be even worse in usual patients in the ICU.

## Conclusion

To respect the principle of autonomy in the informed consent procedure, patients should be able to decide freely without any external pressure whether they agree to continue to participate in a clinical trial at any time. In concordance with our previous paper, about 80% of ICU patients are able to recall their clinical trial participation. However, as hypothesized, the rate of recall of the clinical trial components is very low. We presume that it could be much lower than in patients informed before being admitted to the ICU. Our results reinforce the importance of viewing informed consent as a process and the need for revisiting informed consent several times during an ongoing clinical trial. We suggest that investigators, while the research is conducted., repeatedly repeat clinical trial information to the participants even after their ICU stay. We therefore propose to reassess regularly whether continued participation in a clinical trial is desired by the patient, as recommended previously [11]. Whether encouraging patients to read the informative leaflet and ask questions about the clinical trial improves the informed consent procedure remains to be fully investigated.

## Key messages

• The rate of recall of the clinical trial components (purpose and risks) in patients informed after being admitted to the ICU is very low. It is presumably much lower than in patients who gave their informed consent outside the ICU setting.

• More patients who could recall their clinical trial participation and the clinical trial components had read the informative leaflet or had asked at least one question before signing the informed consent.

• Reconsidering the informed consent procedure repeatedly during an ongoing clinical trial could be useful to respect patients' rights.

• Informed consent to participate in a clinical trial should be considered an ongoing process rather than a single procedure.

## Abbreviations

GCS = Glasgow Coma Scale; ICU = intensive care unit.

## Competing interests

The authors declare that they have no competing interests.

## Authors' contributions

CC participated in the design of the study and the collection, analysis and interpretation of the data and wrote the manuscript. PM contributed to the conception and design of the study and to the analysis and revision of the manuscript. SL was involved in the design of the study and the collection of data. BR conceived and coordinated the study and was involved in the interpretation of data and in revision of the manuscript. All authors read and approved the final manuscript.

## Figures and Tables

**Table 1:** Demographic, anamnestic characteristics and intensive care unit data of the two groups of patients

Characteristic	Complete recall	Incomplete recall	*p*
*n*	14	30	
Demographic and educational data			
Age, years (mean ± SD)	54 ± 22	54 ± 21	> 0.99^b^
Male/female, *n*	9/5	19/11	0.95^c^
Educational status			
Up to junior high school, *n *(percentage)	6 (43)	11(37)	0.89^d^
Intermediate (college), *n *(percentage)	6 (43)	16 (53)	
Academic level or more, n (percentage)	1 (7)	1 (3)	
Not available, *n *(percentage)	1 (7)	2 (7)	
Daily alcohol intake, *n *(percentage)	1 (7)	8 (27)	0.23^c^
ICU and hospital data			
Admission diagnosis, *n *(percentage)			0.77^c^
Abdominal surgery, *n *(percentage)	7 (50)	10 (33)	
Cardiovascular surgery, *n *(percentage)	3 (21.5)	8 (27)	
Trauma, *n *(percentage)	3 (21.5)	9 (30)	
Other, *n *(percentage)	1 (7)	3 (10)	
Emergency admission/elective admission, *n*	7/7	16/14	> 0.99^c^
Admission SAPS II (mean ± SD)	16 ± 10	20 ± 9	0.24^b^
Length of mechanical ventilation^a^, hours (median (range))	0 (0–15)	0 (0–64)	0.47^e^
Length of ICU stay, days (median (range))	3 (2–9)	4 (2–25)	0.43^e^

ICU, intensive care unit; SAPS II, Simplified Acute Physiology Score second version. In the complete recall group, patients able to mention their clinical trial participation and the two clinical trial components; in the incomplete recall group, patients were unable to mention their clinical trial participation or one of the clinical trial components. ^a^None of the patients were intubated at the time of the informed consent procedure; ^b^Student's *t *test; ^c^Fisher's exact test; ^d^χ^2 ^test; ^e^Mann–Whitney *U *test.

**Table 2:** Data at the time of informed consent and the attitude of the patients

Parameter	Complete recall	Incomplete recall	*p*
*n*	14	30	
Data at the time of informed consent			
GCS (mean ± SD)	15 ± 0	15 ± 0	> 0.99^a^
SOFA score (median (range))	2 (1–5)	2.5 (0–9)	0.18^b^
Temperature, °C (mean ± SD)	37.0 ± 0.5	37.2 ± 0.7	0.21^a^
Glycemia, mmol/l (mean ± SD)	7 ± 2	7 ± 3	0.38^a^
Creatinin, μmol/l (mean ± SD)	80 ±± 25	105 ± 78	0.24^a^
Bilirubin, mmol/l (mean ± SD)	13 ± 6	15 ± 6	0.18^a^
Medications			
24 hours before informed consent			
Morphine, *n *(percentage)	2 (14)	11 (37)	0.17^c^
Benzodiazepines, *n *(percentage)	1 (7)	5 (17)	0.65^c^
Vasopressors, *n *(percentage)	0 (0)	0 (0)	
24 hours after informed consent			
Morphine, *n *(percentage)	8 (57)	18 (60)	> 0.99^c^
Benzodiazepines, *n *(percentage)	1 (7)	3 (10)	> 0.99^c^
'Attitude' of the patients			
Read leaflet before consent, *n *(percentage)	11 (79)	17 (57)	0.20^c^
Asked at least one question before consent, *n *(percentage)	8 (57)	6 (20)	0.03^c^
Read or asked, *n *(percentage)	13 (93)	18 (60)	0.03^c^
Read and asked, *n *(percentage)	6 (43)	5 (17)	0.13^c^

GCS, Glasgow Coma Scale; SOFA, Sequential Organ Failure Assessment score. In the complete recall group, patients able to mention their clinical trial participation and the two clinical trial components; in the incomplete recall group, patients were unable to mention their clinical trial participation or one of the clinical trial components. ^a^Student's *t* test; ^b^Mann–Whitney *U* test;^c^Fisher's exact test.

**Table 3:** Value of 'asking one question' and 'reading the informative leaflet or asking one question' to recall the clinical trial

Parameter	OR, complete versus incomplete recall	Sensitivity	Specificity	Positive predictive value	Negative predictive value	Likelihood ratio
Asked one question	5.3 (1.3–21)	0.57 (0.29–0.82)	0.80 (0.61–0.92)	0.57 (0.29–0.82)	0.80 (0.61–0.92)	2.86
Read leaflet or asked one question	8.7 (1.0–75)	0.93 (0.66–1.00)	0.40 (0.23–0.59)	0.42 (0.25–0.61)	0.92 (0.64–1.00)	1.55

OR, odds ratio. In the complete recall group, patients able to mention their study participation and the two clinical trial components; in the incomplete recall group, patients were unable to mention their clinical trial participation or one of the clinical trial components. Figures in parentheses are 95% confidence intervals.

**Table 4:** Patient characteristics, rate of recall and attitude of the patients during the informed consent procedure according to the two investigators.

	Investigator 1	Investigator 2	*p *value
Age of the patients, mean (SD)	48 (22)	56 (20)	0.21
Sex of the patients, male/female	11/4	17/12	0.29
SAPS II of the patients	20 (9)	18 (10)	0.56
Patients with Complete recall, *n *(percentage)	3 (20)	11 (38)	0.38
Patients with recall of study participation, *n *(percentage)	10 (67)	25 (86)	0.14
Patients with recall of the study purpose, *n *(percentage)	4 (27)	16 (55)	0.07
Patients with recall of the study risk, *n *(percentage)	7(46)	14(48)	0.99
Attitude of the patients before consent, *n *(percentage)			
Read leaflet	8 (53)	20 (69)	0.30
Asked at least one question	6 (40)	8 (28)	0.40
Read or asked	10 (67)	21 (72)	0.74
Read and asked	4 (27)	7 (24)	0.99
